# The Philosophy of Crime and Pauperism, with Suggestions of Possible Methods of Prevention

**Published:** 1898-07

**Authors:** F. E. Daniel

**Affiliations:** Austin, Texas


### TEXAS MEDICAL JOURNAL.

ESTABLISHED JULY, 1885.

PUBLISHED MONTHLY.—SUBSCRIPTION $1.00 A YEAR.

VOL. XIV. AUSTIN, JULY, 1898. No. 1.

### Original Contributions.

### The Philosophy of Crime and Pauperism, With Suggestions of Possible Methods of Prevention.

BY F. E. DANIEL, M. D., AUSTIN, TEXAS.

[Address before the Congress of the National Prison Association, at Austin, Texas, December 2, 1897.]

It would be profitless to thrash over beaten straw or to recount an oft-told tale. I will not occupy your time by going over the discussion of threadbare subjects however important; but hope to offer a few thoughts which have at least the merit of originality, and which I trust may be found not unworthy of consideration.

This association has met annually for more than a quarter of a century to discuss prison reform. It is composed of learned judges, brilliant lawyers, gifted divines, skilled physicians and experienced wardens and superintendents of prisons and asylums. They have brought to each congress the fruits of their labors and observations; they have compared views and tallied experiences, and with regard to many of the details of the subject, have reached definite conclusions. In able papers and addresses and discussions by men distinguished no less for their philanthropy than for their learning, every phase of the question has been considered. These papers and addresses have appeared annually in a volume of "Transactions," constituting in itself a treatise on criminology. In brief, everything that can or need be said has been said, and there is a unanimity of sentiment that our penal system is defective in many essentials, and a change is needed. The association is world-famous for its labors. It has earned laurels even in Europe. Members have been the recipients of royal tokens of appreciation, and we recall with pride the honors bestowed by the czar of Russia and the president of the French on our distinguished collaborator, the Hon. C. H. Reeves of Indiana, whose work on "The Prison Question" is a classic.

And yet, so far as my information extends, very little real progress has been made in prison reform, and less in reform in criminal jurisprudence. Results have not been by any means commensurate with the labors of the association or the necessity for change, either in this country or in Europe, where delegates from this association have participated in the labors of international congresses. The commissioner of the London *Chronicle* says:

"I question whether a single reform of the smallest consequence has been introduced since 1878, a period covered, be it noted, by the work of such men as Lombroso and Ferri and the American Prison reformers, and by a series of experiments not less important, perhaps, than those which were responsible for the 'Origin of Species.'"

The legislation necessary to finish off the work—to give form and effect to the conclusions of this body—has not been forthcoming. In business, in every department of life, old methods tried and found unsatisfactory are discarded. Changes are made as progress demands, in the administration of private interests, but our criminal jurisprudence has stood still; it is notoriously slow to assimilate any facts revealed either by science or experience, and our prison methods, demonstrably defective, and injurious in the highest degree, remain unchanged; we are running in the same old rut.

Reforms of the nature of those here proposed are matters of slow growth, of education. Sentiments deep-rooted in the popular mind, the effect of customs long established, have to be overcome, and the necessity of change clearly demonstrated. The work of this body, able and brilliant, is as a sealed book to all but members and a few sympathizers whose attention has been called to it; it is entombed in the volume of "Transactions." I very respectfully suggest that the publication of a periodical devoted to the dissimination of the association's work, for popular distribution, is worthy of consideration as a possible medium of popular education along these lines.

I will not attempt to enumerate the changes which, by a concensus of opinion amongst members, seem to be practically agreed upon as imperatively needed. In many respects it has been shown that our prison methods are not adapted to the ends for which they are ostensibly instituted, or, rather, for the ends they ought to accomplish. I will only mention the evil of the indiscriminate herding together of hardened criminals and first offenders awaiting trial or being sent to the penitentiary; the tender lad, maybe for his first offense, chained to a notorious criminal; the infamous lease system which still disgraces our civilization; the still worse form of enforced idleness in penitentiaries which has been recently instituted in New York with such disastrous results that the first week nine of the unfortunates went mad. Such a system is unworthy an advanced civilization, and in operation practically defeats the aims of the State, if those aims be what a rational concept of crime and criminals suggests, reformation—the cure of the curable (and Professor Smith says that 95 per cent are curable), the restoration to usefulness and to society, and not the certainty of making a confirmed criminal of every offender, under the operation of the definite sentence. Sentiment has greatly changed in late years with reference to crime and criminals, and people are beginning to realize that while there is a distinct criminal class, every person who commits a crime is not necessarily a criminal; but before he is adequately punished in "vindication of the majesty of the law," under the mistaken notion that justice is being satisfied he is certain to become one. "The State pursues a course to harden his heart," says Warden French, "and make him a confirmed criminal." People are beginning to realize, too, that crime is a disease of the social body, to be cured, and not punished; and our prisons should be moral hospitals, and no longer places of sin, degradation, humiliation and punishment, "compulsory schools of crime—the nursery of vice" (says Gen. Brinkerhoff), our penitentiaries "a moral tomb, a sepulchre of dead souls," as Professor Henderson says, "that become a purgatory and at last a hell." It is clear in the minds of just men that any law that makes retaliation and vengeance its end, and regards punishment as synonymous with justice is wrong in conception and hurtful in execution.

Punishment for crime is not justice. The fundamental principle of penal justice away back in the ages was pecuniary compensation—restitution. It is still the underlying principle in law. Vengeance was reserved for the alien and the enemy. At what period in the world's history they were blended—got mixed—I can not say, but we seem to have entirely lost sight of the distinction. The early Jews and the Welsh Britons practiced restitution, and it was also a feature of the Mosaic law. That the State is but the "wider family," as Professor Peabody says, (Chautauqua lectures) and should be, and is, paternal, is a modern awakening to the original meaning of "society," and we should return to first principles. The existing idea of justice seems to be the outcome of the police regulations which Moses found it necessary to make for controlling that turbulent, barbaric race—the rescued Jews in their flight from Egypt—through the wilderness. What family would put to death one of its children for wrong-doing?

Capital punishment, even regarded in the light of vengeance or expiation, is a failure even of that barbarous end. The blow aimed at the malefactor falls, in every case, on innocent heads that ought to be protected, and not infrequently reacts upon the State itself. To the culprit it is but a momentary pang; to his family it means eternal disgrace, want, distress, if not starvation. Take a case which occurred in a neighboring State recently: A man with a wife and five small children dependent upon his labor for support, killed another man similarly circumstanced. It was his first offense. The State executed that man and both families became a charge upon the taxpayers. That was modern justice. The "majesty of the law" was vindicated, and two widows and ten orphans were made paupers, outcasts, and doubtless, finally, criminals. The pretext that this man, because he had the misfortune to kill another was an incurable criminal, a danger to society, and must, therefore, be eliminated, is the sheerest nonsense, yet it is the only grounds upon which it can be pretended to be justified; it is clearly vengeance.

People ore begining to realize these things, and the sentiment should be cultivated. They are beginning to understand that two wrongs do not and can not make a right. That capital punishment fails to check crime is demonstrated by the rapid increase of crime of every kind everywhere, despite law and lynching. There is more than a suspicion that it promotes it by a kind of "suggestion." That it does not "protect society" is shown by the growth of mob law, and that it should be abolished as worse than useless, barbarous, inexpedient, inhuman, inexcusable from whatever standpoint regarded, is, I believe, the consensus of those who have given intelligent thought to the subject.

This brings us face to face with the desideratum of first importance, the problem to be solved primarily, and on the solution of which depends whether or not this association's labors have been vain and fruitless; on its solution depends the future usefulness of this body and the solution of all other problems connected with the subject. That problem is, how can the conclusions of this body be effectually impressed upon those entrusted with the making and the mending of the law? By what means can the work done heretofore be incorporated into our jurisprudence, that the reforms so much needed and insisted upon may be put into practical operation? The real obstacle lies deeply imbedded in our socio-political system and is irremediable. In past ages, in all ancient nations, even amongst savage tribes, the making of the laws has been entrusted to the wise men. In our day and generation it is left to a heterogeneous body (not class) of men, thrown haphazard together every two years by popular choice, and chosen for any other consideration too frequently, than that of wisdom or fitness, young men predominating as a rule. Young and old and middle-aged, black and white, learned and unlearned, farmer, mechanic, lawyer, merchant, Christian, Jew, Gentile and pagan —hardly any two of them of like habits of thought or educated in the same direction or to the same degree; men who are wise only in their own conceit, too often, and who are notoriously averse to being advised. The young American statesman as we see him in legislative halls of some states, is a finished product, whom the wisest in this body can not enlighten on any subject, either of science, politics or state government; he knows it all. Nevertheless, the problem of prison reform can never be solved practically without the co-operation of state legislatures. To secure that, then, is the first essential; hence the difficulty.

But, gentlemen, our criminal laws and prison regulations all have to do with existing criminals. As important as is the question how best to handle them, it is overshadowed and eclipsed by the fact that criminals are being produced faster than they can be killed, cured, cared for, reformed or disposed of; increasing in a ratio out of all proportion to population. We are continually enlarging our penitentiaries and asylums, without realizing, seemingly, that the time must come when these classes will exist in such numbers as to be beyond control or accommodation. We have to maintain now, penitentiaries for an aggregate of 100,000 convicts, according to our distinguished president, and it "requires an army larger than that of the United States to protect society from the lawless element," and, he adds significantly, that if society can not be protected "it demonstrates the necessity for a stronger form of government." In the Texas reformatory, according to Superintendent McGuire, there are 800 boys; "225 where 100 ought to be," he adds.

It is true, the subject of prevention has received great, careful and earnest attention at the hands of this association; but I do not know of any legislation designed to, in the least, interfere with the increase of criminals; or any statute directed to any of the many sources of undesirable population; neither towards regulating marriage, as earnestly and logically urged by Judge Reeves (see Prison Question) or to change the environment of the thousands of youths who annually graduate from those kindergartens of crime, the streets and slums of our cities, two sources recognized as prolific of crime, heredity and environment; no statute that will arrest the out-put of criminals from our jails and penitentiaries after definite sentence, the raw material having gone in as first offenders.

I believe with Professor Ferri (Criminal Sociology) that "the volume of crime will not be materially diminished by codes of criminal laws, however skillfully they may be constructed; but by an amelioration of the adverse individual and social conditions of the community as a whole. Crime is a product of those adverse social conditions, and the only way of grappling with it is to do away, as far as possible, with the causes from which it springs." What are those causes and how can they be done away with?

Legislation can do much to ameliorate that condition of society; but the defect lies too deep; it is too intimately interwoven with the warp and woof of our social fabric to be reached and eradicated. Society is maladjusted, the effect of unwise and unjust laws, and it is a moral certainty that it will never adjust itself. What, then, is the alternative? What the logical conclusion?

Of the numerous factors that enter into the genesis of crime, a sad feature of the maladjustment and adverse condition just mentioned, I believe that idleness is the most prolific. That "idle hands make work for the devil" is a proverb as true as it is homely. That idle hands make work for the police and for the criminal courts is certain. Our jails are largely recruited from the streets, where boys are allowed to grow up untaught. Warden Cassidy says "industrial education is the only thing that prevents crime," and Pastalozzi "found the remedy for vice and crime to be education." Director Clark of the trades school at Elmira says: "To have the resources of gaining an honest living materially strengthens the power to resist the impulse to secure a dishonest one." Mr. Spencer says: "Labor and honesty go hand in hand, as do idleness and crime." Hall Caine says: "The morals of a nation are closely bound up in their material condition; teach the boys the facts of life and the spirit of chivalry." Gen. Brinkerhoff says: "Men in idleness rapidly deteriorate, both physically and mentally;" work is essential to human health and happiness. I quote Professor Sam G. Smith: "There is nothing more invigorating to character than hard work, successfully and adequately paid, for successful work not alone produces the means by which the desires of the body and mind are gratified, but is in itself a restraining of unwholesome desires and a means by which the will is enthroned and the conscience given voice." "Need and idleness increase crime in bad years, the wages being too small for virtue."

The government, therefore, that permits its youth to grow up in ignorance and idleness; that makes laws of such unjust and uneven character that under their operation one class grows richer and the other poorer; that entail a condition under which a man is not able to find employment at any kind of labor, is responsible for the crime and pauperism of that country and ought to make restitution. Who and what will constitute, twenty years hence, the great horde of convicts in place of those on hand today, puzzling this body what to do with them? The babes of today and the unborn. It should be prevented. The government should see that they are trained and equipped for citizenship and not for criminals and paupers; and there will be many more than now.

"It is God's law," says Mr. Brockway, "that unless a man works he shall not eat." That is a very unjust law when one can not get the work to do.

It would be interesting to know how many idle men there are today in these United States—men willing to work, anxious to work, whose necessities compel them to work, in default of which they hunger and their families starve? It is said that Debs offered to muster 50,000 honest laboring men, idle and hungry, in Chicago in one day. Mr. George said that in New York there were 68,000 idle working-men, a-hunger. How many Coxey took to the nation's capitol in rags and hungry I do not know, but it must have been a touching sight. If other cities can come up with a pro rata approximating the above the total is something fearful to contemplate, and may well give us pause. It is a lamentable state. Beyond all doubt the great army of criminals is recruited largely from this mass of idle humanity, as are also the army of insane, the hordes of paupers; the list of suicides is swollen, the "grand army of tramps" is kept upon a war footing, and last, but not least, the cause of anarchy is aided, abetted and strengthened.

If, then, my premises are correct: if ignorance and idleness are the cause, or in part the cause, of the growth of crime and pauperism, the logical remedy is to be looked for in industrial schools and employment for the idle population. If the enforced idleness be the result of unwise and unequal laws the government is responsible, and in justice and equity, as well as from considerations of policy, of humanity, should find the employment for the idle and the schools for the ignorant. "All are agreed," says Gen. Brinkerhoff, "that upon discharge of a prisoner he should be cared for, aided in securing employment," and it is insisted that prisoners should be taught a useful trade to enable him to make a living when discharged, and it seems the general sentiment of this body that the State shall find it for him. "The discharged prisoner," says Chaplain Bradshaw, "is friendless, nameless, suspected by society, shadowed by human sleuth hounds, blackmailed by former companions and is driven to join the criminal ranks." He is ostracized, disfranchised and shunned, and his alternative is a dishonest life or starve. Who and what made him so? The State, by neglect, and when, in consequence of that neglect he committed an offense against the laws, by a course of severity calculated to make him commit other offenses, to be, in short, a criminal.

And here I will ask, as has been asked a thousand times; if, as a matter of policy, or it is advisable from whatever standpoint to do these things for the convict and the ex-convict, is it not a thousand times more obligatory on the State, more rational and just to do the same for the honest laborer and the street boys, who, for want of it will securely become a criminal, or insane, suicide, pauper or anarchist? It is a shame and a reproach that any man able and willing to work should be hungry, much less starve, for want of work to do. Is it any wonder that there is discontent, that there is a growing sentiment of anarchy?

In light of the foregoing, would it not be a wise thing for the general government to at once institute some great public work upon which every idle man could find steady employment at living wages? The general government annually expends thousands, nay, millions of dollars upon an ineffective levee system in the vain endeavor to prevent the overflow of the Mississippi river, whereby millions in money and time are lost by the destruction of growing crops, stock and cattle. When an overflow threatenes, more dirt is piled on, until, at New Orleans, the river level is several feet above the streets. Invariably a crevasse occurs at some weakest point (there is always a weakest point), the pressure is removed, and the levees below, and crops out of the way of the escaping waters are saved. Nature here points us to a remedy which we would do well to adopt. Make the crevassees permanent, or rather imitate them by tapping the river, including its chief tributaries (the engineers say the overflows are due primarily to the Ohio and Missouri) at the most suitable points, but instead of letting the water find its way out, conduct it to the nearest adjacent water course. Let these floodgates be so arranged that when the pressure reaches the danger point it will flow out and through these canal, thus distributing it over a larger area; the effect will be the same, whether the water be returned by the collateral stream to the Mississippi lower down or not. It is believed that a system of canals as suggested could be constructed that would prevent these destructive overflows; and while it would be a gigantic undertaking, it would be completed in time, while the patching up of the useless levees goes on forever. And the cost of the work, compared to the sums sunk in the vain efforts to confine the water to the river bed, to say nothing of the added losses of crops and stocks, etc., would be insignificant. Aside from the great economic value of such a work, it would be an efficient prophylaxis of crime, pauperism, suicide, insanity and anarchy. By relieving the necessities of the idle masses, it would in a great measure quiet the discontent now threatening open revolt. Imagine the effect of an announcement that tomorrow morning the United States government would give employment to every idle man in

America at $1.50 a day. In 1873, during the labor riots and strikes in New York, the mayor, Hon. Fernando Wood, said: "We will build Central Park." It was built; the men were put to work and the effect was like oil on troubled waters. In my deliberate judgment, such a course would in a few years tell most favorably on the statistics of our defectives.

Should the views herein respectfully submitted for your con sideration meet with favor, I would suggest the appointment of a commission to appear personally before congress and lay the matter before them, and that in each State there be a committee on legislation, the duties of each committee to be to present to legislative bodies, in the briefest but most forcible manner, the views of this association touching the several questions upon which conclusions have been reached, and ask for the legislation necessary to give them form and effect. Ask congress to give the idle men work; to abolish capital punishment and the lease system, the definite sentence, and insist that in each State industrial schools be established, attendance upon which shall be compulsory. No boy should be permitted to grow up in ignorance and idleness. There are 50,000 children loose in the streets of New York because of lack of school accommodations. It would be true economy in the end to expend millions upon the foundation of such schools, and as much more upon a system of canals as outlined above, or some similar work, the saving of the vast sums lost by floods and the millions wasted on a futile levee system being in favor of the first mentioned.

It is very evident that something should be done; some effort made in the name of humanity for the relief of suffering in the country; and that some steps should be taken to arrest the ever-increasing influx of undesirable population, the growth of crime and pauperism and insane and suicide, is demanded by every consideration of public safety, economy and common sense. Dr. Wines says that our census statistics are not correct. True, they are not accurate, but they are approximate, and as there is no other criterion we must use them.

According to the census of 1890, the last published, there were in the United States 83,328 prisoners; (Gen. Brinkerhoff says 100,000, now) against 6737 in 1850. In 1850 there was one prisoner to every 3347 population. In 1890 there was one prisoner to each 756 of population. In 1890 there were 106,257 insane, or 1697 to each 100,000, against 765 in 1860. In 1890 there were 95,571 idiots and feeble minded, 1526, against 602 to each million in 1860. In 1890 there were 50,411 blind, both eyes, 805 against 403 to the million in 1860; 41,283 deaf and dumb, or 659 against 408 to the million in 1860; 73,045 paupers, ratio not given. And these classes are increasing in ratio out of all proportion to the increase of population, and each decade with a steady progression.

When we reflect on these facts and realize, as Gen. Brinkerhoff, our president, says, that it requires an army larger than that of the United States to protect society from the lawless element, that gaunt hunger stalks abroad in the land; when we read that in New York, and presumably in other cities, hundreds of idle workmen stand in line all night, and will fight for place in line, waiting for the dole of stale bread given them at intervals from the bakery; that Hungarian laborers, as Mr. George said recently in a speech, are working in the mines of Pennsylvania for from 5 cents to 20 cents per day, and hundreds ready to take their places; that these men have been shot down without provocation; that the cry of the oppressed is stifled by injunctions backed by the artillery of the United States army, do we wonder that there is discontent, wonder at the growth of anarchy? Men are not going to sit down and quietly starve. History shows that when they cannot get bread they shed blood. If an American citizen, however humble, is arrested in a foreign land, the whole army will be brought into requisition, if necessary, to protect him; it may be made a casus belli. In times of general distress in foreign lands, in India, China, Russia, the sympathies of the American congress are moved to send shiploads of food and donations of money to their relief. Our government looks on with apparent indifference and unconcern at the distress of thousands of our citizens, her citizens at home, who are starving for the want of work, or are working at starvation wages. At the country's call the people sprang to arms and struck the shackles from 4,000,000 black slaves. Today, under the operation of unwise immigration laws, whereby the refuse of Europe have come into the country to take the work that belongs to the American laborer, untold thousands of freeborn American citizens have been reduced to a condition worse than that of the black slaves, in all but the name. The slave never starved nor his family went unfed. The master received the proceeds of his labor—as now—but in return for it gave his slave comfortable quarters and sufficient wholesome food and warm clothing. In bad weather the slave was excused from outdoor work and his children were cared for. In sickness the slave and his family had the best medical skill. It is not human nature to love a government under whose laws a condition like this is possible, and the immigration laws are not alone to blame. That law which makes sacred from taxation the earnings of the millionare's money violates every principle of justice and is enough to drive the masses to revolt.

> "Ill fares the land to hastening ills a prey,
> Where wealth accumulates and men decay."

Have we a realizing sense of the true condition of the laboring class? I beg to quote from a letter from a lawyer, a former citizen of Texas, now resident in a large city in the far west:

"This State is in the grasp of Eastern mortgage companies. They virtually own every prominent building in the city, as the money loaned on them is beyond the power of payment. Not five families in a hundred own their homes. The papers have daily increasing notices of foreclosure of mortgages on all kinds of real estate, and the proceeds go east. Wages are very low. Cedar shingles go begging at $1 per 1000, and the wheat, constantly falling, is mostly in the hands of the farmers. Labor and capital are very bitter towards each other, and the revolutionary feeling is intense. The condition of the masses is pitiable in the extreme. Women and children are doing the work while the men are idle, the former being cheaper, having taken their places. Taxes, $1,250,000 to 60,000 population, and the condition is not much better in other cities."

Revolution is a thought uppermost in men's minds. Grave, thoughtful men predict it, works of fiction are written to give voice to the sentiment that it is the logical alternative of the situation. English papers are speculating on the probable downfall of the American republic. When we recall the prediction of Macaulay in 1857, that in the twentieth century this republic would be ravaged and plundered by barbarians, the product of our own institutions, does it not look like a prophesy dangerously near fulfillment? Will our lawmakers not be warned by lessons of the past? Is there no teaching in the history of the French commune, the fate of Louis XVI and Marie Antoinette, by which even a republican government can profit? The disciples of John Wickliffe are abroad in the land. "The Mad Priest of Kent," preaching the equality of man, and the extinction of classes survives today in the Debs, the Powderlys, the Sovereigns, the Georges. Have we forgotten Watt Tyler and his 100,000 men of Kent? Tyler failed. He gave his life, but it was the beginning of the end of serfdom in England. The villainage or serfdom of his day is paralleled by the condition of the American laboring classes of today.

Every breeze that sweeps across the continent bears on its wings the mutterings of restless discontent and tales of suffering. There is an apathetic indifference to the condition of desperation born of idleness and hunger and close bordering on revolt. No one heeds the premonitions of threatened trouble. In the cities the feast of Belshazzar is daily spread, the worship of Baal is uninterrupted, God is forgotten or defied. The handwriting is upon the wall, but no man heeds it. The cry of distress, the discontent, the strikes, the riots, the labor meetings where capital is denounced as the enemy of labor, these things are all unheeded; but, like the mutterings of distant thunder, they surely presage the coming of a storm.

